# Physical Exercise Potentials Against Viral Diseases Like COVID-19 in the Elderly

**DOI:** 10.3389/fmed.2020.00379

**Published:** 2020-07-03

**Authors:** Sandra Amatriain-Fernández, Thomas Gronwald, Eric Murillo-Rodríguez, Claudio Imperatori, Alexandre Francisco Solano, Alexandra Latini, Henning Budde

**Affiliations:** ^1^Faculty of Sport Sciences and Physical Education, University of A Coruña, A Coruña, Spain; ^2^Department of Pedagogy, Faculty of Human Sciences, Medical School Hamburg, Hamburg, Germany; ^3^Department of Performance, Neuroscience, Therapy and Health, Faculty of Health Sciences, Medical School Hamburg, Hamburg, Germany; ^4^School of Medicine, Anahuac Mayab University, Mérida, Mexico; ^5^Cognitive and Clinical Psychology Laboratory, Department of Human Science, European University of Rome, Rome, Italy; ^6^Laboratório de Bioenergética e Estresse Oxidativo - LABOX, Departamento de Bioquímica, Centro de Ciências Biológicas, Universidade Federal de Santa Catarina, Florianópolis, Brazil

**Keywords:** physical exercise, physical activity, viral diseases, coronavirus, COVID-19, immune response, high-risk groups, elderly

## Introduction

In the last few months, we have been living through an epic public health threat around the globe due to the spread of a novel coronavirus (SARS-CoV-2) that causes coronavirus disease 2019 or COVID-19 (1). COVID-19 is clinically characterized by fever, cough, fatigue, incapacity to breathe, pneumonia/other respiratory tract symptoms, kidney failure, neurological symptoms and even death (1–7). Recent publications have shown that COVID-19 impairs immune system response by severely compromising the number and function of T cells, especially Natural Killer (NK) cells, by increasing the levels of blood C-reactive protein (CRP) and pro-inflammatory cytokines and causing atrophy of spleen and lymph nodes, along with reduced lymphocytes in lymphoid organs (2–7). This immune dysregulation had a fatal outcome mainly in individuals with pre-existing medical conditions and elderly patients (2, 4, 7).

According to the United Nations, there were 703 million people aged 65 years or older worldwide in 2019, which implies that about 10% of our world population is at higher risk for negative prognosis under COVID-19 infection. Aging is characterized by several changes, including exacerbated inflammatory responses mediated by the innate immune system with reduced capacity to protect against infections, cancer and wound healing, leading to more severe consequences of bacterial and viral infections and reduced response to vaccination (8). This pro-inflammatory status renders older individuals susceptible to tissue-damaging immunity and chronic inflammatory diseases. Therefore, interventions that can prevent or retard the decline of immunocompetence would have a considerable clinical and public health impact on this parcel of the population. In this scenario, the regular practice of physical activity and physical exercise has been widely prescribed, including for elderly people, since it favors anti-inflammatory status, promoting healthier aging and reducing all-cause mortality (9).

## Chronic and Acute Physical Exercise Interventions and Their Effects on the Immune System

Physical activity is defined as any bodily movement produced by the contraction of skeletal muscles, including sports, leisure activities, dancing, walking, and physical exercise (10). Physical exercise, meanwhile, is defined as any planned, structured and purposeful physical intervention. Therefore, physical exercise is physical activity, but physical activity is not necessarily physical exercise (11). Both can be chronic when practiced for a long period of time, or acute when only practiced once (12).

It has been extensively demonstrated that the regular practice of physical exercise (chronic exercise or exercise training) at a moderate intensity [64–76% of the maximal heart rate (13)] induces the activation of several signaling pathways, conveying a sustained anti-inflammatory and antioxidant response. Some of the positive effects of chronic exercise on the immune system are related to increased T cell proliferative capacity, neutrophil function, and cytotoxic activity of NK cells (14). For example, 6 months of moderate-intensity aerobic exercise (15–40 min *per* session, 3 times *per* week) provoked a significant increase in the T cell number in the blood of older adults (15); 12 weeks of moderate-intensity walking training (30–40 min, 5 days *per* week) showed enhanced NK cells activity in elderly women (16); and male volunteers practicing moderate-intensity physical exercise had increased neutrophil phagocytotic activity in an age-associated manner (17). Additionally, chronic exercise has also been shown to enhance the immune response against bacteria and viruses, which might constrain or delay immunological aging or immunosenescence (8). Active muscles release cytokines, which are able to counterbalance pro-inflammatory mediators, *i.e*., IL-1β and IL-18, and stimulate the production of IL-1rα and IL-10 interleukins that enhance the anti-inflammatory facet of the immune response (18).

Acute bouts of physical exercise, like a 30-min walk at a moderate intensity (19) or a brief exercise like rapidly ascending 260 stairs (moderate-high intensity) (20) have also been shown to enhance immune system activity by increasing the antipathogen activity of tissue macrophages in parallel with leukocytosis with higher numbers of neutrophils, NK cells, cytotoxic T cells, and immature B cells. Acute bouts of resistance exercises, like 45 min of a moderate-intensity strength session, showed an increase of the immune system response in aging individuals (21). This kind of exercise that increases muscular strength has been demonstrated to reduce metabolic and cardiovascular diseases and is one of the most important stimuli for fighting osteoporosis in aging individuals (22). This immune-strengthening effect contributes to deconstructing the key pillars of the “open window” theory, which hypothesizes that a single acute and vigorous activity might temporarily impair the immune response, increasing the risk of an opportunistic infection (23). The transient and time-dependent redistribution of immune cells to peripheral tissues might actually represent a heightened state of immunosurveillance and competence driven by a preferential mobilization of cells to areas more susceptible to infection after exercise (e.g., lungs and gut) (24–26).

It has been shown that moderate-intensity cardiovascular exercise executed three times per week for 4 months, prior to viral exposure, improved influenza vaccination responses, with extended duration of antibody levels in older adults (27). These enhanced responses emphasize the importance of exercise during a global pandemic, as it was already suggested (28), since both a single session of acute exercise or a repetition of the exercise over time boosts the immune system independently of age, physical fitness, or the presence of pathologies (29).

Considering that exercise is a drug-free treatment, a specific dosage and administration time for achieving maximal effectiveness will be required (30). The phenomenon called hormesis is defined as an adaptive response of cells and organisms to a moderate (usually intermittent) stress (31). This phenomenon explains both, the benefits that exercise interventions (acute or chronic, with a moderate to high intensity) have in our organism, as well as the negative effects caused by overtraining, like the deregulation of the inflammation processes and a decrease in the ability to maintain homeostasis or homeodynamic regulation (32–34).

## Physical Activity and its Effects on the Immune System

Moderate weekly physical activity (defined as at least 180 min per week of walking, occupational/volunteer physical activities) was found to correlate with lower levels of inflammatory markers, like the cytokine tumor necrosis factor alpha (TNF-α) and CRP in a cross-sectional study with a sample of 3,075 subjects aged 70–79 years (35). Habitual physical activity was also associated with the maintenance of neutrophil migratory dynamics in a sample of 211 healthy older adults (67 ± 5 years) (36).

In addition to the benefits of different kinds of physical exercise, it has been shown that a physically active lifestyle might also delay immunosenescence (37), as well as reduce infection risk in the elderly (38). An active lifestyle can also limit adipose tissue accumulation and therefore prevent the development of obesity (39), which represents a state of accelerated aging characterized by low-grade chronic inflammation (37). In fact, the accumulation of visceral fat has been linked to impaired T cell proliferation and function (40). It is known that adipose tissue suffers a growth in response to prolonged overnutrition, sedentary behavior and aging and turns out to be a major cause of chronic inflammation. The pro-inflammatory status contributes to the onset of damaging diseases such as insulin resistance, diabetes, cardiovascular diseases, musculoskeletal disorders, and some cancers (endometrial, breast, ovarian, prostate, liver, gallbladder, kidney, and colon) (41), which themselves were shown to be risk factors for more severe consequences of the SARS-CoV-2 outbreak (2–7). All of this is relevant especially in light of the affirmed 31 million adults in the US aged 50 or older who are inactive according to the Center for Disease Control and Prevention. The World Health Organization (42) calculates that 1 in 4 adults worldwide does not meet the global recommendations for physical activity per week (a minimum of 150 min a week of moderate-intensity aerobic activity, or at least 75 min of vigorous-intensity aerobic physical activity, or an equivalent combination of both). Several interventions have been tried for years to delay aging of the immune system in the elderly with disappointing outcomes, due to high costs of development and administration or for lack of adhesion due to complicated logistics (37). In this scenario, the practice of physical activity and physical exercise appears as a potentially cheap and drug-free tool that boosts the immune response without adverse side effects.

## Molecular Pathways

The current understanding of molecular pathways underlying the effects of the regular practice of physical exercise on health includes the activation and interplay of three major systems, namely the immune response, bioenergetics and resistance to oxidative stress [for a review see da Luz Scheffer and Latini (43)]. Sirtuins, a widely distributed family of proteins responsible for the regulation of many fundamental biological processes, including longevity and health span have been suggested to be the master regulators of the beneficial effects of exercise (44). Sirtuins are either mono-ADP ribosyltransferases or nicotinamide adenine dinucleotide (NAD)-dependent histone deacetylases activated by cellular stress, such as that induced by acute or chronic exercise that reactivates cellular defenses and increases cell metabolism and repair-activities (44). Once activated, sirtuins modify histones, transcription factors and cytoplasmic proteins. For example, by deacetylating PGC-1α (proliferator-activated receptor-γ coactivator-1) (45) and the FOXO (class O of forkhead box) family of transcription factors, sirtuins modulate mitochondrial biogenesis and stimulate the expression of key antioxidant enzymes, including catalase, manganese superoxide dismutase and thioredoxins, respectively (46). In this scenario, it has been demonstrated that exercise increases the activity of sirtuins in the heart and skeletal muscle among other tissues, not only in young people or adults but also in the elderly (47, 48). More recently, it was also demonstrated that sirtuins control the production of pro-inflammatory cytokines in innate immune cells, the type of cells engaged in viral-immune responses. The activation of macrophages, a main source of pro-inflammatory cytokines secreted in response to infection and environmental stress, was shown to occur through two of the major pro-inflammatory pathways in the immune response: NF-κB and AP-1 pathways (49, 50). It was also demonstrated that sirtuins are implicated in the differentiation of activated T cells into CD8^+^ T cells, which are lymphocytes responsible for killing host cells infected with pathogens (51). Thus, it seems promising to use physical exercise as a non-pharmacological intervention for increasing resistance to a variety of immune-related diseases.

## Discussion

The aging of the immune system seems to be responsible for several comorbidities presented in the elderly, and T cells are highly relevant for adaptive immune responses (8, 52). Viruses like SARS-CoV-2 can quickly compromise the number and function of T cells and promote an increased level of pro-inflammatory cytokines in the blood, which might have fatal outcomes in people with pre-existing medical conditions and elderly patients (2–4, 6). Thus, both people with existing chronic pathologies and older populations are at higher risk of responding worse to the viral infection due to their higher susceptibility to different infectious diseases, autoimmune diseases, cancer, obesity, and/or a generally sedentary lifestyle. Furthermore, these populations will also have a worse response to vaccination when compared with younger or healthier individuals (21, 25, 27, 37).

**Figure 1:**
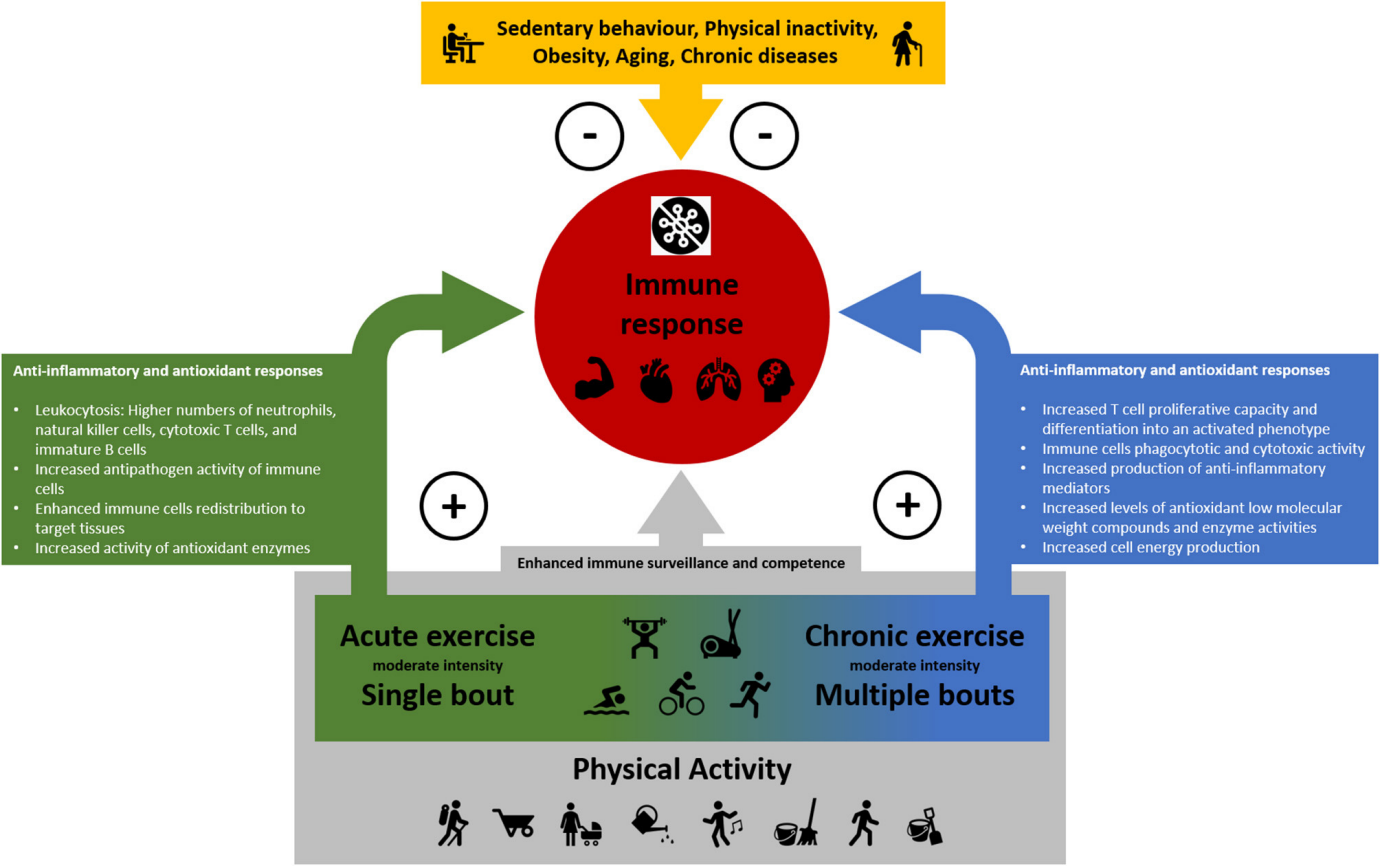
The positive impact of physical activity, acute and chronic physical exercise and the suppressive effect of sedentary behavior, physical inactivity, obesity, chronic diseases, and aging on the immune response.

The benefits of the physical exercise-induced immune response, including increased antipathogen activity, enhanced recirculation of anti-inflammatory cytokines, and leukocytosis are relevant for fighting viral infections (14, 19, 20, 53). At a molecular level, sirtuins might be one of the key regulators behind the beneficial effects of exercise during the aging process (34, 44). Thus, all forms of increased energy expenditure induced by muscle contractions lead to immune-enhancing effects (see Figure 1). Even if someone were inactive in the past, now might be a good time to start exercising in order to be well-prepared to fight infections. Due to the benefits that an active lifestyle has been shown to have on the immune system, it can be suggested that physically active individuals, including the elderly and people with chronic pathologies, are more likely to have a mild progression of viral diseases like COVID-19.

## Author Contributions

SA-F, AL, and HB designed and conceived the paper. SA-F, AL, HB, TG, EM-R, CI, and AS critically revised the manuscript. All authors contributed to the article and approved the submitted version.

## Conflict of Interest

The authors declare that the research was conducted in the absence of any commercial or financial relationships that could be construed as a potential conflict of interest.
